# Supplementary material for: Antioxidant Phytocomplexes Extracted from Pomegranate (Punica granatum L.) Using Hydrodynamic Cavitation Show Potential Anticancer Activity In Vitro
Source: Antioxidants (Basel). 2023 Aug 4;12(8):1560. doi: 10.3390/antiox12081560 (PMC10451776; doi:10.3390/antiox12081560)
Supplement: Supplementary file 1 [file antioxidants-12-01560-s001.zip › antioxidants-2503782-supplementary.pdf]

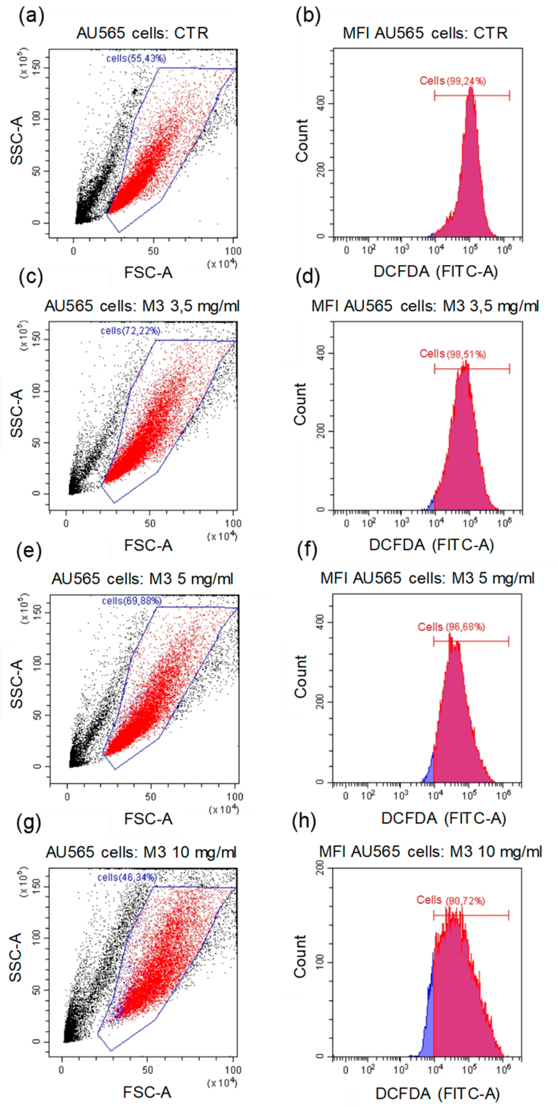

Figure S1. Gating strategy by flow cytometry to assess the reactive oxygen species in AU565 treated with M3 extracts. Representation of the distribution of AU565 alive cells by scatter plot (FSC *vs* SSC): (**a**) CTR; (**c**) M3 extract 3.5 mg/mL; (**e**) M3 extract 5 mg/mL; (**g**) M3 extract 10 mg/mL. Representative histogram of mean fluorescence intensity (MFI) of DCFDA (FITC-A) to assess reactive oxygen

species (ROS) in AU565 alive cells: (**b**) CTR; (**d**) M3 extract 3.5 mg/mL; (**f**) M3 extract 5 mg/mL; (**h**) M3 extract 10 mg/ml. The representations shown are of a representative experiment of three performed.

Table S1. Secondary metabolites in pomegranate samples. The content of total phenols, flavonoids, and tannins of M1-M5 fractions from crude, lyophilized and aqueous pomegranate extracts obtained by spectrophotometric analysis was reported. The mean values (n=3) are to be considered as µg GAE/mg FW for phenolics, µg QE/mg FW for flavonoids, µg CE/mg FW for tannins, and µg of each anthocyanin/mg FW for anthocyanins. Legend: SD, Standard Deviation; Pg3G, Pelargonidin 3-glucoside; Pt3G, Petunidin 3-glucoside.

Samples		Phenolics		Flavonoids		Tannins		Anthocyanins													
		Mean	SD	Mean	SD	Mean	SD	Cyanidin		Delphinidin		Pg3G		Peonidin		Malvidin		Pelargonidin		Pt3G	
								Mean	SD	Mean	SD	Mean	SD	Mean	SD	Mean	SD	Mean	SD	Mean	SD
Crude	M1	**0.162**	0.014	**2.123**	0.056	**0.378**	0.026	**0.161**	0.010	**0.120**	0.007	**0.326**	0.010	**0.111**	0.002	**0.060**	0.005	**0.198**	0.008	**0.372**	0.019
	M2	**0.216**	0.021	**2.403**	0.045	**0.415**	0.021	**0.214**	0.013	**0.156**	0.005	**0.426**	0.013	**0.146**	0.004	**0.074**	0.002	**0.261**	0.010	**0.473**	0.028
	M3	**0.211**	0.042	**2.638**	0.062	**0.401**	0.024	**0.227**	0.005	**0.176**	0.012	**0.448**	0.027	**0.157**	0.006	**0.087**	0.003	**0.284**	0.017	**0.516**	0.021
	M4	**0.191**	0.014	**2.702**	0.085	**0.490**	0.025	**0.244**	0.010	**0.185**	0.011	**0.464**	0.019	**0.154**	0.006	**0.094**	0.004	**0.280**	0.020	**0.524**	0.029
	M5	**0.201**	0.028	**2.806**	0.017	**0.402**	0.016	**0.221**	0.013	**0.170**	0.009	**0.421**	0.029	**0.154**	0.009	**0.086**	0.005	**0.250**	0.017	**0.499**	0.035
Lyophilized	M1	**0.206**	0.021	**2.195**	0.395	**0.392**	0.020	**0.091**	0.005	**0.073**	0.004	**0.156**	0.012	**0.061**	0.004	**0.027**	0.001	**0.102**	0.006	**0.216**	0.009
	M2	**0.269**	0.019	**2.243**	0.079	**0.398**	0.024	**0.170**	0.009	**0.124**	0.009	**0.348**	0.028	**0.120**	0.007	**0.048**	0.003	**0.218**	0.015	**0.380**	0.023
	M3	**0.387**	0.208	**2.746**	0.023	**0.384**	0.019	**0.221**	0.018	**0.162**	0.006	**0.160**	0.003	**0.152**	0.006	**0.076**	0.005	**0.274**	0.022	**0.499**	0.015
	M4	**0.255**	0.021	**2.630**	0.085	**0.379**	0.015	**0.220**	0.009	**0.163**	0.008	**0.454**	0.018	**0.157**	0.006	**0.077**	0.003	**0.279**	0.006	**0.507**	0.041
	M5	**0.196**	0.021	**2.890**	0.034	**0.366**	0.011	**0.186**	0.009	**0.144**	0.012	**0.361**	0.018	**0.127**	0.010	**0.071**	0.004	**0.244**	0.007	**0.427**	0.026
Aqueous	M1	**0.080**	0.017	**1.756**	0.099	**0.661**	0.040	**0.005**	0.000	**0.178**	0.007	**0.527**	0.042	**0.182**	0.011	**0.038**	0.003	**0.330**	0.010	**0.528**	0.037

	M2	**0.129**	0.009	**1.931**	0.021	**0.551**	0.028	**0.282**	0.020	**0.189**	0.013	**0.550**	0.022	**0.196**	0.016	**0.044**	0.002	**0.362**	0.011	**0.597**	0.018
	M3	**0.129**	0.004	**2.175**	0.028	**0.677**	0.047	**0.129**	0.006	**0.218**	0.011	**0.619**	0.025	**0.216**	0.006	**0.048**	0.004	**0.397**	0.020	**0.655**	0.026
	M4	**0.153**	0.004	**2.410**	0.120	**0.719**	0.022	**0.338**	0.014	**0.233**	0.009	**0.637**	0.038	**0.229**	0.009	**0.051**	0.002	**0.425**	0.026	**0.687**	0.034
	M5	**0.153**	0.009	**2.210**	0.035	**0.722**	0.036	**0.287**	0.011	**0.193**	0.012	**0.579**	0.041	**0.197**	0.008	**0.038**	0.002	**0.359**	0.022	**0.613**	0.025

Table S2. Phenolic and flavonoid compounds detected in pomegranate extracts by HPLC-DAD. Concentrations of secondary metabolites present in the fractions (M1-M5) of the crude. lyophilized and aqueous extracts were reported. Results were expressed as μg per mg of plant fresh weight.

EXTRACT	FRACTION	Mean/Standard deviation	Gallic acid	3-Hydroxytyrosol	Vanillic acid	Epicatechin	Rosmarinic acid	4-Hydroxybenzoic acid	Chlorogenic acid	Caffeic acid	Syringic acid	*p*-Coumaric acid	Resveratrol	Salicylic acid	Quercetine-3-O-glucoside	Myricetin	Quercetine	5.7-Dimethoxycoumarin	Genistein	Kaempferol	1.1-Dimethylallyl caffeate	Chrysin	Caffeic acid phenethyl ester
CRUDE	**M1**	Mean	**0.00**	**451.70**	**51.91**	**384.87**	**1.93**	**61.67**	**208.75**	**23.37**	**32.45**	**12.95**	**48.84**	**120.00**	**155.96**	**4.88**	**0.74**	**0.00**	**0.00**	**0.32**	**0.00**	**0.00**	**0.00**
		Stnd Dv	0.00	27.10	3.11	15.39	0.15	4.93	16.70	1.87	0.97	0.52	2.93	8.40	6.24	0.24	0.04	0.00	0.00	0.02	0.00	0.00	0.00
	**M2**	Mean	**0.00**	**478.89**	**58.92**	**576.77**	**2.24**	**68.70**	**263.73**	**26.53**	**40.50**	**16.50**	**66.25**	**188.72**	**215.92**	**5.66**	**0.17**	**0.00**	**0.00**	**0.25**	**0.00**	**0.00**	**0.00**
		Stnd Dv	0.00	28.73	4.12	34.61	0.09	2.75	15.82	1.59	1.22	0.50	1.32	7.55	8.64	0.34	0.01	0.00	0.00	0.02	0.00	0.00	0.00
	**M3**	Mean	**0.00**	**542.42**	**65.20**	**606.15**	**1.05**	**83.38**	**142.65**	**29.36**	**45.35**	**17.62**	**71.51**	**176.88**	**223.11**	**2.66**	**0.39**	**0.00**	**0.00**	**0.37**	**0.00**	**0.00**	**0.13**
		Stnd Dv	0.00	37.97	4.56	18.18	0.03	4.17	5.71	1.17	1.81	1.06	4.29	10.61	13.39	0.11	0.01	0.00	0.00	0.03	0.00	0.00	0.01
	**M4**	Mean	**0.00**	**556.45**	**33.47**	**292.41**	**2.40**	**129.56**	**95.30**	**15.07**	**5.21**	**3.12**	**18.53**	**162.99**	**182.96**	**6.07**	**0.14**	**0.26**	**0.00**	**0.38**	**0.00**	**0.00**	**0.29**
		Stnd Dv	0.00	33.39	2.01	20.47	0.17	5.18	3.81	0.60	0.10	0.12	1.11	3.26	12.81	0.49	0.01	0.01	0.00	0.02	0.00	0.00	0.01
	**M5**	Mean	**0.00**	**465.07**	**34.44**	**282.96**	**2.70**	**121.13**	**100.91**	**15.51**	**5.69**	**2.98**	**8.49**	**148.95**	**177.52**	**6.81**	**0.14**	**0.47**	**0.00**	**0.36**	**0.00**	**0.00**	**0.28**
		Stnd Dv	0.00	18.60	1.38	11.32	0.16	7.27	6.05	0.62	0.23	0.12	0.34	11.92	14.20	0.54	0.01	0.01	0.00	0.01	0.00	0.00	0.01
LYOPH ILIZED	**M1**	Mean	**0.00**	**394.59**	**42.17**	**328.23**	**1.06**	**73.58**	**81.75**	**18.99**	**27.90**	**10.70**	**27.58**	**13.17**	**154.61**	**2.68**	**0.25**	**0.00**	**0.00**	**0.35**	**0.00**	**0.00**	**0.00**
		Stnd Dv	0.00	27.62	2.95	13.13	0.03	5.89	4.91	0.76	0.84	0.21	1.65	0.92	12.37	0.11	0.01	0.00	0.00	0.01	0.00	0.00	0.00

	M2	Mean	0.00	374.20	46.15	340.11	2.70	49.05	194.89	20.78	29.46	10.72	204.19	135.93	178.78	6.82	0.31	0.00	0.00	0.29	0.00	0.00	0.00
		Stnd Dv	0.00	29.94	1.85	20.41	0.08	1.96	7.80	1.25	2.06	0.43	12.25	5.44	12.51	0.48	0.02	0.00	0.00	0.02	0.00	0.00	0.00
	M3	Mean	0.00	475.93	57.05	400.76	2.11	86.01	228.93	25.69	36.78	11.39	236.11	125.85	189.48	5.33	0.30	0.00	0.00	0.36	0.00	0.00	0.00
		Stnd Dv	0.00	28.56	3.99	32.06	0.06	2.58	9.16	0.77	1.47	0.68	16.53	5.03	15.16	0.11	0.01	0.00	0.00	0.03	0.00	0.00	0.00
	M4	Mean	0.00	425.99	53.45	382.06	2.03	51.98	227.06	24.07	32.73	11.41	28.41	118.15	144.38	5.13	0.35	0.29	0.00	0.33	0.00	0.00	0.00
		Stnd Dv	0.00	21.30	3.21	21.78	0.14	2.08	6.81	1.93	1.31	0.34	1.70	7.09	4.33	0.21	0.02	0.02	0.00	0.03	0.00	0.00	0.00
	M5	Mean	0.00	419.82	60.33	535.67	2.46	160.67	130.62	27.17	38.44	13.97	56.13	4.93	165.84	6.22	0.18	0.42	0.00	0.24	0.00	0.00	0.15
		Stnd Dv	0.00	8.40	4.22	37.50	0.17	8.03	3.92	1.09	1.54	0.56	1.68	0.20	9.95	0.37	0.01	0.03	0.00	0.02	0.00	0.00	0.01
AQUEOUS	M1	Mean	0.00	0.00	64.43	460.36	0.00	217.31	156.26	29.02	20.28	0.00	13.31	137.80	203.48	0.00	0.00	0.00	0.00	0.00	0.00	0.00	1.46
		Stnd Dv	0.00	0.00	2.58	18.41	0.00	17.38	12.50	1.74	1.22	0.00	0.27	6.89	8.14	0.00	0.00	0.00	0.00	0.00	0.00	0.00	0.06
	M2	Mean	0.00	0.00	5.82	198.07	1.30	38.53	86.32	2.60	2.24	0.00	13.94	160.06	236.70	3.28	0.00	0.00	0.00	0.00	0.00	0.00	0.00
		Stnd Dv	0.00	0.00	0.35	11.88	0.04	1.16	2.59	0.10	0.11	0.00	0.98	11.20	13.02	0.29	0.00	0.00	0.00	0.00	0.00	0.00	0.00
	M3	Mean	0.00	0.00	79.04	642.98	1.72	89.02	338.73	35.57	26.62	0.00	14.14	172.69	254.85	4.26	0.00	0.00	0.00	0.00	0.00	0.00	0.00
		Stnd Dv	0.00	0.00	5.53	45.01	0.12	3.56	13.55	1.42	1.86	0.00	0.57	6.91	10.19	0.17	0.00	0.00	0.00	0.00	0.00	0.00	0.00
	M4	Mean	0.00	0.00	76.34	611.47	1.46	113.26	358.02	34.37	29.33	0.62	14.66	143.47	275.08	3.69	0.00	0.00	0.00	0.00	0.00	0.00	0.00
		Stnd Dv	0.00	0.00	4.58	36.69	0.09	9.06	28.64	1.37	1.17	0.02	0.29	4.30	11.00	0.26	0.00	0.00	0.00	0.00	0.00	0.00	0.00
	M5	Mean	0.00	0.00	59.64	461.34	1.77	95.52	135.77	26.83	7.12	2.29	13.31	118.40	258.02	4.52	0.00	0.00	0.00	0.00	0.00	0.00	0.00
		Stnd Dv	0.00	0.00	2.39	18.45	0.12	3.82	8.15	1.34	0.36	0.07	0.40	3.55	5.16	0.36	0.00	0.00	0.00	0.00	0.00	0.00	0.00
